# Coffee Silverskin as a Potential Ingredient for Functional Foods: Recent Advances and a Case Study with Chocolate Cake

**DOI:** 10.3390/foods13233935

**Published:** 2024-12-05

**Authors:** Adriana S. Franca, Emiliana P. Basílio, Laís M. Resende, Camila A. Fante, Leandro S. Oliveira

**Affiliations:** 1 Programa de Pós-Graduação em Ciência de Alimentos (PPGCA), Universidade Federal de Minas Gerais, Av. Antônio Carlos, 6627, Belo Horizonte 31270-901, MG, Brazil; emilianabasilio@gmail.com (E.P.B.); lais.maia.resende@gmail.com (L.M.R.); camilafante@farmacia.ufmg.br (C.A.F.); leandro@demec.ufmg.br (L.S.O.); 2 Departamento de Engenharia Mecânica (DEMEC), Universidade Federal de Minas Gerais, Av. Antônio Carlos, 6627, Belo Horizonte 31270-901, MG, Brazil

**Keywords:** coffee by-product, coffee silverskin, functional food, dietary fiber, antioxidants, sensory analysis

## Abstract

Coffee silverskin (CS) is a by-product of the coffee roasting process that is known for its potential as a fiber source with antioxidant properties. Therefore, this study aimed to provide an overview of the latest research on CS as a potential ingredient for functional foods and to evaluate the effect of adding different amounts of CS on the functional and sensory attributes of chocolate cakes. The addition of CS increased the total dietary fiber content, antioxidant capacity and the contents of extractable and non-extractable phenolics in the cakes. The evaluated sensory attributes were color, smell, taste, texture and overall impression, and they were evaluated according to a 9-point hedonic scale. Internal preference maps were obtained based on the results from acceptance and “intention to buy” tests. In general, the cakes with lower coffee silverskin content (2.6% and 3.6%) had a similar level of acceptance and the cake with 4.6% coffee silverskin content was the least accepted. The most important attributes were taste and overall impression, corresponding to “like slightly” and “like moderately” for the cakes that had better acceptance. Nonetheless, even with the lowest amount of added CS (2.6%), the produced cakes could be regarded as antioxidant fiber sources (with fiber content above 3 g/100 g), thus confirming the potential of CS as a functional food additive.

## 1. Introduction

Roughly one-third of the food produced for human consumption is wasted, and for every ton of produced food, an equal amount of waste or by-products is also generated, posing significant negative environmental and economic impacts [1]. It is estimated that the global production of food waste and by-products will increase by over 200 million tons by 2050 [2], and thus efficient waste management strategies are necessary.

Coffee is one of the most popular beverages around the world and one of the most important commodities traded worldwide [3]. The global production in the 2023/2024 crop years was over 10 million tons [4]. Coffee silverskin (CS), the integument that covers the raw coffee bean, is a solid residue that is generated during coffee roasting. It is detached and removed by heated air during this process and collected by cyclone separation [5]. Several studies have shown that CS and other coffee processing by-products can be employed in a wide variety of applications, including acoustic insulation of buildings, fuel, animal feed, polymeric materials and as ingredients for pharmaceutical and food products [3,6,7,8]. A recently published comprehensive review on the current applications of coffee silverskin is available in the literature [9].

CS has been reported to have high dietary fiber content and has been established as a potential source of bioactive compounds with antioxidant potential and health benefits; such compounds include caffeine, chlorogenic acid and melanoidins [10,11]. Its beneficial health effects are directly related to its fiber composition, physicochemical properties and associated antioxidant compounds. These correspond to polymeric or small polyphenol molecules that are linked or trapped by the food matrix, the so called non-extractable phenolics (NEPAs) or macroantioxidants [12]. These compounds are not extracted from food by the organic aqueous solvents that are traditionally used for determination of total phenolics, and thus are not considered in most analyses. These substances are not released during chewing, or by the acidic pH of the stomach or by the action of digestive enzymes, and thus reach the colon intact along with the dietary fibers, conferring specific properties to these fibers, given their intrinsic antioxidant capacity [13]. Although some studies have indicated that CS is a potential source of antioxidant substances, none have evaluated the NEPA contents.

In view of the aforementioned factors, the aim of this study was to provide an insight into the potential of CS as a functional food ingredient. A brief overview of the coffee processing steps and corresponding by-products is presented, followed by data on the chemical composition and recent food-related applications of CS, focused on the development of food products. Finally, a case study on the application of CS as an ingredient in chocolate cakes is presented. The effect of adding different amounts of CS on the functional and sensory attributes of chocolate cakes is discussed, including, for the first time in the literature, the evaluation of non-extractable phenolics.

## 2. Coffee Processing Overview

**Figure 1:**
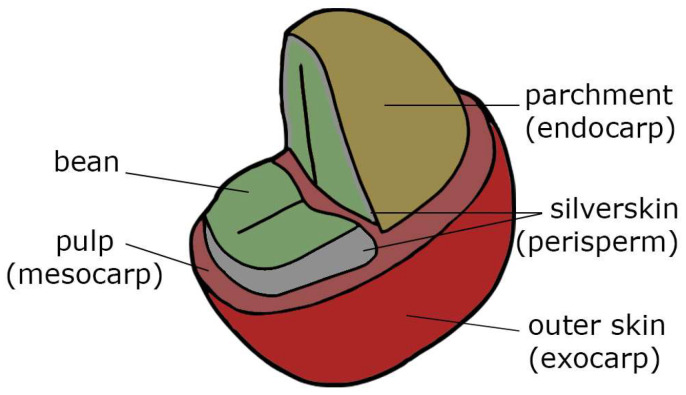
Schematic view of the coffee fruit.

The coffee plant belongs to the genus Coffea of the Rubiaceae family, with the two commercially relevant species being *Coffea arabica* and *Coffea canephora*. The coffee fruit or so-called coffee cherry (Figure 1) usually has red or yellow peel (exocarp) when ripe. The mesocarp, a gelatinous pectic pulp, encloses the mucilage layer that is firmly adhered to parchment (endocarp). This parchment encapsulates two coffee seeds (coffee beans) that are covered by the perisperm—a thin, closely fitting sclerotic layer called silverskin. 

**Figure 2:**
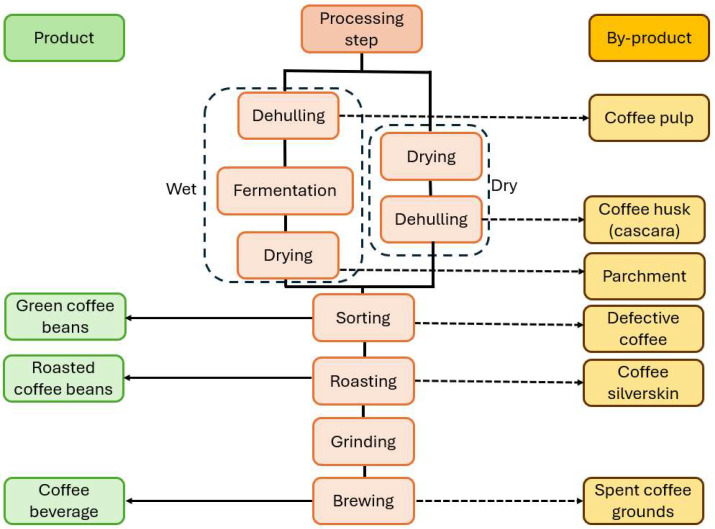
Summarized view of the main steps of coffee processing.

Once the coffee fruits have been harvested, they undergo so-called “primary processing”, a series of operations that culminate in the separation of the coffee beans from the fruit. A schematic overview of the general steps involved in primary processing is shown in Figure 2. Processing can be dry, semi-wet or wet. In dry processing, the harvested fruits are usually spread over a large concrete patio and left to dry in the sun for a period of 15 to 30 days, depending on climate conditions. If necessary, mechanical dryers are employed to remove the final percentage points of moisture. Also, in farms where the construction of large patios is not feasible, coffee cherries are dried directly in mechanical dryers. A less common method is drying coffee cherries over a suspended wire mesh, with no need for frequent raking and turning of the fruits to ensure even drying and prevent bacterial damage, since the atmospheric air flows both over and under the suspended bed of cherries [5]. After drying, the coffee fruits are dehulled to separate the beans from the dry pulp and parchment. During semi-wet processing, on the other hand, dehulling takes place before drying, with the exocarp and mesocarp being mechanically removed from the freshly harvested coffee cherries using a pulping machine, in which the fruits are pressed between a screw conveyor and a perforated stationary cylinder metal wall. The applied pressure allows for the pulp to burst from the coffee fruits and pass through the wall perforations. Immature fruits pass through without bursting and are also separated. The beans (with the parchment still attached) are placed in patios for sun-drying. A mechanical dryer may also be used to attain the desired moisture level. During the wet process, after the fresh fruits have been dehulled, the parchment coffee is fermented to remove any residual pulp and mucilage, then it is washed and sun-dried [5]. Afterwards, the parchment is removed from the dry beans by mechanical dehulling. The dry coffee beans are cleaned and graded (by size, density and color) and finally stored in standard 60 kg bags for later commercialization. The color-sorting procedure facilitates the separation of low-quality or defective beans (i.e., immature or green, sour and black beans), which are also sold at a lower price [5]. 

Once the coffee beans are commercialized, they undergo the “secondary processing” phase, which will result in the final products—usually roasted and ground coffee. During roasting, the coffee beans are submitted to controlled moderate pyrolysis (hot air heating at 200 to 220 °C), until the desired roasting degree is attained. In this stage, the coffee beans go through several chemical reactions and physical changes that ultimately give them the desired flavor and aroma. During roasting, the silverskin is loosened, given the attrition between coffee beans when tumbling inside the roaster; it is then released with the hot air and collected in a cyclone [5,6]. Afterwards, the beans are cooled, left to rest and then ground (or not) and packed, at which point they are ready to be commercialized for domestic or commercial purposes. In summary, the main by-products that are generated during primary and secondary coffee processing include coffee husks and pulp, parchment and silverskin. Further processing (soluble coffee or beverage production) will result in the final by-product: spent coffee grounds.

## 3. Coffee Silverskin

Coffee silverskin (CS) is the only by-product resulting from the roasting process, and for each 60 kg bag of coffee, approximately 2.5 kg of CS is generated [14], amounting to over 400,000 tons of CS being discarded annually worldwide. Considering the significant quantities being produced, and the consequent environmental implications, many studies have focused on devising alternative uses for coffee silverskin [15,16]. The range of applications is quite diverse, and some recent examples include the use of CS as fillers for polymeric composites [17], and in the production of biohydrogen [18] and adsorbents for removal of pollutants [19] among others. Table 1 presents a few selected recent (from 2023 to 2024) studies that have evaluated CS for different purposes).

A few recent studies have focused on the use of CS in polymeric materials, either as fillers, aiming to improve the polymer mechanical, thermal, and barrier properties, or as additives that can improve specific properties such as antioxidant characteristics [20,21,22,23]. Although addition of untreated CS can lead to decreases in hardness and elongation given the adhesion between polymer and fiber and the formation of hydrogen bonds [20], the use of alkali treatments was shown to increase both tensile strength and flexibility [20,22]. Also, the presence of bioactive substances, including caffeine, chlorogenic acids, cafestol and kahweol, among others, has prompted the use of CS as an active ingredient, with applications in active packages [22] and cosmetics [24]. In this case, the preparation of extracts rather than the direct use of ground CS seems to be more effective. Finally, as is common for agricultural wastes in general, some studies aimed at finding alternative uses for CS have focused on applications such as solid fuels, soil amendment and animal feed [25,26,27]. Nonetheless, the complex chemical makeup of coffee and respective by-products points towards a wider range of applications.

Besides the previously mentioned applications, many studies have focused on the use of CS as a food additive, given the potential health-related benefits that are associated with coffee [9]. A recent review pointed out that caffeinated coffee consumption in the US was inversely correlated to several health disorders, including cardiovascular disease, type 2 diabetes, hepatocellular carcinoma endometrial cancer, melanoma, and nonmelanoma skin cancer [28]. Several studies have confirmed that moderate daily consumption of coffee can be associated with positive health effects [29], which is attributed to coffee’s rich phytochemistry, as it includes bioactive substances such as caffeine and chlorogenic acids, among others [30]. These bioactive compounds are found not only in coffee itself, but also in the solid wastes or by-products resulting from coffee processing [31], including coffee silverskin (CS). Reports on anti-inflammatory, antioxidant and antiaging effects [9,32], antibacterial activity [33], as well as the antidiabetic potential [34] of CS are available. Also, recent studies indicate that CS is a source of active substances that can combat muscle atrophy and metabolic syndrome [35]. 

In the following section, a discussion of the main aspects of the chemical composition of CS is presented, which will provide a better understanding of its recent food-related applications.

### 3.1. Composition

A comprehensive overview of the chemical composition of CS was presented by Narita [15]. The average composition consists of dietary fiber (50–60%), protein (16–19%), lipids (1.56–3.28%) and minerals (7%). Dietary fibers correspond to carbohydrate polymers that are neither digested nor absorbed in the small intestine and have been extensively studied due to their beneficial health effects, including attenuation of blood cholesterol, improvements in large bowel function and weight maintenance, reduction in glycemic response and reduced risk of coronary heart diseases [36]. The dietary fibers in CS can be divided into insoluble (IDF, 53–56%) and soluble (SDF, 9–11%), with slightly higher IDF/SDF ratio values reported for Robusta in comparison to Arabica coffees [37]. Monosaccharide analysis highlighted glucose (50.8–56.2%) and xylose (26.3–30.4%) as the main constituents of IDF polysaccharides. The high glucose content is mainly attributed to cell wall polysaccharide cellulose, while both glucose and xylose are derived from xyloglucans. The dominant monomers in SDF are rhamnose (26.9–33.5%), arabinose (18.2–24.4%), mannose (17.1–18.5%), and galactose (13.7–8.1%), with the potential original polysaccharides being type II arabinogalactans, galactomannans, and glucomannans [37]. 

A detailed overview of the mineral composition of CS was recently published [38]. CS contains high levels of calcium (1080 mg/100 g) and potassium (972 mg/100 g), suggesting possible exploitation in the production of calcium ingredients for food or pharmaceutical applications. While calcium is an essential element for various physiological functions, potassium plays a key role in the maintenance of blood pressure, as well as in the muscular and nervous systems. Other minerals present in significant amounts include magnesium (257 mg/100 g), sodium (110 mg/kg) and sulfur (51.9 mg/kg). Magnesium has been reported to play several roles in the normal functioning of the human body, thus confirming CS as a potential source of minerals [38]. Regarding microminerals, of a total of 23 elements, the most abundant were iron (238 mg/kg), aluminum (89.0 mg/kg), manganese (46.7 mg/kg), copper (37.9 mg/kg), zinc (31.9 mg/kg) and boron (26.1 mg/kg). These are important micronutrients that play important health-related roles, affecting red blood cell formation and fatigue reduction (iron), bone health maintenance, oxidative stress modulation, connective tissue formation (copper and manganese) and protecting the immune system and cells from oxidative stress (zinc), among other bodily functions [38].

A recent study reported the occurrence of 45 peptides in CS that could be associated with proteins involved in several metabolic pathways. Analysis of the protein fraction confirmed the presence of serine proteases, carboxypeptidase and neprosin, which could be associated with potential positive action of CS extracts in the digestion process [38]. Other proteins involved in carbohydrate metabolism and polyphenol oxidation were also detected. In the amino acid profile, glutamic acid, aspartic acid and leucine were present Iin higher amounts. Nonetheless, considerable amounts of the essential amino acids leucine, valine, phenylalanine, isoleucine, threonine, histidine and lysine were also detected [39]. CS is also a source of vitamins B2 (riboflavin, 0.18–0.20 µg/g) and B3 (niacin, 2.51–3.07 µg/g) [38].

The chemical profile of CS has been extensively investigated from the point of view of bioactive compounds, with the most significant being caffeine (0.8–1.25%), chlorogenic acid (1–6%), trigonelline and melanoidins [39,40]. Caffeine, the world’s most popular stimulant and psychoactive substance, is the most abundant active compound found in brewed coffee and is one of the most valuable compounds in CS, given its wide range of pharmacological applications and potential benefits for human health in moderate doses. It has been associated with a reduction in the risk of several diseases, including Parkinson’s, Alzheimer’s, and type II diabetes, among others [41]. Its beneficial effects also include increased alertness, as well as anti-inflammatory and immunosuppressant actions. Chlorogenic acid, the most abundant isomer of caffeoylquinic acid, is an important and biologically active dietary polyphenol with antioxidant activity and significant positive health-related effects, possessing antibacterial, hepatoprotective, cardioprotective, anti-inflammatory, anti-microbial, anti-hypertension and anti-obesity properties, among others [42]. Melanoidins are Maillard reaction products that form during roasting as products of reactions between the amino groups of amino acids, proteins, or vitamins, and the carbonyl group of reductive sugars or oxidized lipids [9]. 

Given that CS undergoes thermal processing (roasting), a recent study raised concern about some possible carcinogens that can be formed during roasting, such as acrylamide, furfuryl alcohol or hydroxy methyl furfural. Nonetheless, such heat-induced contaminants were detected in rather low concentrations [37].

### 3.2. Recent Food-Related Applications

The nutritional profile of coffee silverskin, especially its abundance in dietary fiber, proteins, and antioxidants, has prompted some studies on its use as an ingredient in food formulations, including breads, biscuits and beverages [9]. Its recent applications also include meat, yogurts, candies, cookies, cereals, breads and cakes [43,44,45,46,47].

A recent study evaluated the feasibility of partial substitution of animal fat with CS on chicken patties [43]. Ground CS was added in increasing amounts. The patties were cooked on a hot plate (without using oil or fat) and their physicochemical, textural, and sensory properties were evaluated. Replacing animal fat with CS led to decreases in moisture content and retention, water activity, pH and luminosity (e.g., an increase in darkness). Regarding the textural parameters, hardness increased compared to the control, while resilience, cohesiveness and springiness decreased. Sensory parameters were negatively affected when the added CS amount was above 2%. Bertolino et al. [44] added both Arabica and Robusta CS into cow whole-milk yogurt to increase its nutraceutical value. Addition of Arabica CS yielded the highest CGA content and the strongest antioxidant activity, while Robusta CS contained the highest amount of caffeine content. It was observed that the bioactive compounds remained bioaccessible during the digestion process, with variations in properties and bioaccessibility of the yogurt depending on the coffee species and on the amount of CS added.

Guglielmetti and collaborators [45] added CS aqueous extracts to gluten-free bread formulations. The extract acted as a natural colorant, providing the typical appearance of wholemeal bread. Adding CS increased the fiber, protein and total extractable phenolics content, resulting in significant improvements in antioxidant capacity. Sensory tests indicated that the developed bread formulation maintained its sensorial quality, and thus was suitable for celiacs and possessed the potential to reduce the risk of gastrointestinal disease related to oxidative stress. Furthermore, in vitro digestion results showed that the bioaccessibility of sugars decreased, while the opposite was observed in terms of antioxidants. Another study evaluated the effect of adding CS hydroalcoholic extract (30% ethanol) on the chemical, physical, microbiological, structural, and sensory attributes of gummy candies [46]. Addition of the CS extract led to increases in bioactive content and antioxidant activity of the candies. Also, CS-enriched candies presented better sensory characteristics in comparison to the control. All parameters were deemed satisfactory even after 120 days of storage, confirming CS’s potential to improve both the functional and sensory quality of confectionary products.

A few recent studies evaluated the potential of CS and its extract as a functional ingredient in cookies. Gocmen and collaborators [47] employed ground CS as a flour substitute in cookie formulations. CS supplementation increased the phenolic content, antioxidant capacity, and in vitro bioaccessibility of antioxidants. Sensory evaluation indicated that adding up to 5% CS could improve functional properties without having significant effects on color perception, taste, texture and product acceptance in general. A more recent study [48] employed both ground CS and its ethanolic/aqueous extract in cookie formulations. Use of the CS extract provided increases in phenolic content and antioxidant activity, and the prepared cookies presented adequate acceptability. Use of ground CS, on the other hand, resulted in significant improvements in functional properties, including an increase in fiber content. However, the cookies presented lower sensory acceptability, with an intense, bitter flavor and aftertaste, a dry and rough texture and toasty smell. The authors recommended the evaluation of new strategies to improve sensory characteristics, such as the incorporation of cocoa powder to help mask unpleasant sensory attributes. CS was also recently employed in extruded cereals, providing increases in both protein and dietary fiber contents of these food products [49].

Ates and Elmacı [50] employed CS as a fat replacer in cake formulations. Adding CS increased the bitterness, hardness and chewiness of the cakes and decreased their springiness and cohesiveness. CS submitted to hot water treatment (10 min in boiling water) was also evaluated. The authors concluded that water-treated coffee silverskin (WCS) could be used to substitute up to 30% of the fat content in the cake, increasing its fiber content without significantly affecting its sensory characteristics in comparison to the control cake. However, water treatment led to a significant decrease in antioxidant activity, with similar values between the control cake and the one with 30% WCS. Even though it was concluded that there was a need to submit CS to water treatment in order to improve cake acceptance, such treatment basically cancels out the antioxidant effect of this residue, so in the present study, we decided to employ CS without any treatment in a chocolate-flavored cake, as suggested by Dauber et al. [48]. Our hypothesis was that chocolate should be able to mask some of the bitterness, allowing both the fiber and the antioxidant content of the cakes to be increased while maintaining acceptable sensory characteristics.

### 3.3. Case Study: CS as a Potential Functional Ingredient in Chocolate Cake

#### 3.3.1. Materials and Methods

##### Materials 

Coffee silverskin, CS (Arabica species, dry processed coffee), was donated by a local roaster (Sigma-Aldrich, Luz, MG, Brazil). Chocolate-flavored cake formulations were purchased from a local market in Belo Horizonte, MG, Brazil. 

The employed reagents were diethyl ether, potassium sulfate, selenium oxide, cupric sulfate, sulfuric acid, boric acid, sodium hydroxide, hydrochloric acid, iron (III) chloride, acetone, butanol, ethanol, methanol, sodium carbonate (Synth, SP, Brazil); alpha amylase, 1,1-diphenyl-2-picrylhydrazyl radical (DPPH), Folin–Ciocalteu reagent, gallic acid, pan-creatin, pepsin, and 2,4,6-Tri (2-pyridyl)-s-triazine (TPTZ) (Sigma-Aldrich, São Paulo, SP, Brazil).

##### CS Preparation and Characterization 

CS was ground (Cadence grinder model MDR301, Cadence, Caxias do Sul, RS, Brazil), sieved (D < 0.5 mm) and used without further processing. The moisture, ash, fat and protein content of CS were determined by AOAC methods: 925.10, 923.03, 945.16 and 968.06 [51], respectively. The carbohydrate content was calculated by subtracting the protein, fat, ash and moisture content from 100 g of CS. Total, insoluble and soluble dietary fiber were quantified by the enzymatic gravimetric method proposed by Asp et al. [52] using the enzymes alpha amylase, pepsin and pancreatin. 

Two CS extracts were prepared for total extractable phenolics (TEPs), DPPH and FRAP analyses. The methanol–acetone extract was prepared according to the method described by Resende et al. [53]. In summary, the CS (1 g) was placed in test tubes and sequentially extracted with 40 mL of methanol (50% *v*/*v*) and 40 mL of acetone (70% *v*/*v*). After each extraction, the mixture was centrifuged at 3500 rpm for 15 min. After that, the supernatants were combined, and distilled water was added to achieve a total volume of 100 mL. The ethanol extract was prepared as described by Ballesteros et al. [54], with modifications. The CS (1 g) was placed in test tubes and extracted with 35 mL of ethanol (60% *v*/*v*) and heated during 30 min in a water bath with magnetic agitation at 60–65 °C. Afterwards, the extract was centrifuged at 3500 rpm for 20 min, the supernatant was transferred to a volumetric flask, and the total volume completed to 50 mL with distilled water.

In our work, phenolic compounds were considered to be the only reducing compounds present in CS, so TEPs were quantified by the Folin–Ciocalteu method as described by Resende et al. [53], with the only difference being the calibration curve range, from 20 to 100 µg/mL gallic acid. In summary, 5 mL of Folin–Ciocalteu reagent was mixed with 1 mL of extract and 4 mL of sodium carbonate solution. The resulting solution was stirred and then left to rest in the absence of light (120 min). Absorbance values were measured at 765 nm using a UV/Vis spectrophotometer (Micronal AJX 1900, Micronal, São Paulo, SP, Brazil). The results are expressed as gallic acid equivalents (mg GAE per g of dry matter) based on a calibration curve built employing gallic acid. The absorbance calibration curve (R^2^ = 0.93) was: y = −0.1871 + 0.0073x, with y representing the absorbance and x the GAE concentration.

Non-extractable phenolics (proanthocyanidins, NEPA) were evaluated according to Zurita et al. [55], with the modifications described by Resende et al. [53]. Residues obtained from the extracts prepared for TEPs were dried (35 °C, 16 h) and subsequently reacted with butanol solution acidified with hydrochloric acid (95:5 *v*/*v*) containing FeCl_3_ (100 °C, 1 h). The remaining material was centrifuged, and the supernatants were recovered. Absorbance readings were taken at 450 nm and 550 nm, and the sum of both values was plotted against NEPA concentration, with polymeric proanthocyanidin concentrate isolated from carob pod being used as a standard. The results were expressed as mg NEPA/100 g flour.

The antioxidant activity of DPPH was evaluated as described by Brand-Williams et al. [56], with modifications. Methanolic solutions of DPPH and its extracts were mixed in different proportions and (0.06 mM) were mixed to 0.1 mL of solutions of the extracts in methanol (3:1, 1:1, 1:3, 1:7 *v*/*v*). The solutions were incubated for 1 h in the dark at room temperature. The samples were analyzed in a UV-Vis spectrophotometer (Micronal AJX 1900, Micronal, São Paulo, SP, Brazil) at 515 nm. Variations in absorbance were observed until stabilization was achieved. The amount of sample (mg) needed to decrease 50% (mL) of the initial DPPH concentration (IC50) was obtained by linear regression. The antioxidant activity was determined via a FRAP assay that was carried out according to the procedure described by Resende et al. [57]. Samples extracts were reacted with FRAP solution (acetate buffer, TPTZ and FeCl_3_) at 37 °C for 30 min and absorbance values measured at 595 nm. The results were expressed as µmol Fe2SO_4_/g, based on a calibration curve employing ferrous sulfate.

##### Cake Preparation and Characterization 

The cake formulations contained sugar, flour (wheat and rice), hydrogenated vegetable oil, powdered cocoa, modified starch, salt, natural flavoring, caramel colorant IV, baking powder (sodium bicarbonate, aluminum phosphate and calcium phosphate) and xanthan gum. The composition information provided by the manufacturer (based on a 33 g portion) was as follows: 26 g carbohydrate, 1.1 g protein, 3.7 g fat, 134 mg sodium and 0 g dietary fiber.

The cakes were prepared using a commercially available cake mixture, with the mixture being replaced with increasing amounts of CS, resulting in four formulations: one without CS (F0—control), and the remainder with added CS in amounts ranging from 20 (F1) to 35 g (F3), as detailed in Table 2. The amount of CS was determined based on preliminary tests to ensure that even the smallest amount would be enough to characterize the cake as a fiber source. According to the Brazilian Legislation [58], foods considered to be sources of dietary fiber are those that contain at least 3 g of fiber per 100 g/mL of prepared food or at least 2.5 g of dietary fiber per portion.

The cakes were prepared according to the manufacturer’s instructions: eggs and milk were added to the mixture and mixed for 1 min, and the batter was poured into aluminum pans and baked in an electric oven (180 °C for 40 min). After baking, cakes were removed from pans and left to cool at room temperature. They were then placed in plastic bags and stored at room temperature.

The prepared cakes were evaluated in terms of total, insoluble and soluble dietary fiber, extractable and non-extractable phenolics, and total antioxidant capacity (DPPH and FRAP), according to the methodologies described under the CS Preparation and Characterization subheading. For these specific analyses, only the control (F0) and the formulations with the lowest (F1) and highest (F3) CS amounts were tested.

##### Sensory Analysis

The sensory analysis evaluated the cakes’ acceptance in a single session. A total of 90 tasters, who were potential consumers aged between 18 and 55 years (21% men; 79% women) were asked to evaluate the cake samples with respect to their color, smell, taste, texture and overall impression. Each person received a brief explanation before the session with respect to the sensory parameters and the use of the hedonic scale (1: disliked extremely, 2: disliked very much, 3: disliked moderately, 4: disliked slightly, 5: neither liked nor disliked, 6: liked slightly, 7: liked moderately, 8: liked very much, 9: liked extremely). Preference maps were drawn from the results of acceptance tests [59]. All participants in the sensory analysis signed an informed consent form. The research was approved by the University Ethics Committee (Process 85563718.1.0000.5149).

##### Statistical Analysis

Analyses were performed in triplicates (except for TEPs, n = 5) with mean and standard deviation. After evaluation of normality, data were statistically analyzed by ANOVA and Tukey’s tests, and a Kruskal–Wallis non-parametric test was employed for NEPA, with 95% confidence (*p* < 0.05). Sensory data were evaluated using SensoMaker software (Version 1, 2024) and the other data were analyzed using IBM SPSS Statistics software, version 19. 

#### 3.3.2. Results and Discussion

##### CS Characterization 

The proximate composition of CS was determined to be as follows: 16.4 ± 0.7 g/100 g protein, 8.5 ± 0.1 g/100 g ashes, 6.4 ± 0.6 g/100 g lipids and 69 g/100 g carbohydrates. The protein values were in the same range as those described in the literature reports, whereas both ashes and lipids were slightly higher. The soluble and insoluble fiber content was determined to be 4.7 ± 1.3 and 63.6 ± 0.4 g/100 g, respectively. The significantly higher quantity of insoluble fibers was expected due to the high amounts of cellulose, hemicellulose and lignin present in CS [60]. 

The results related to the amounts of phenolics and antioxidant potential of CS are displayed in Table 3. The total extractable phenolics varied depending on the employed solvent, with a mixture of methanol and acetone being more efficient than ethanol. The phenolics present in CS correspond mostly to caffeic and chlorogenic acids [61]. The values obtained in this study are in the same range as reported in the literature, with differences mostly associated with the extraction solvent. Reported values range from 400 mg GAE/100 g (methanol 40%) [62] up to 1900 mg GAE/100 g (deep eutectic solvent using ultrasound-assisted extraction) [57]. The NEPA content ranged from 236 to 309 mg/100 g, with the higher value obtained via ethanol extraction, in an amount similar to that reported for several fruit peels, including nectarine and pear [63]. This is the first study that has addressed NEPA content in coffee silverskin. Among the non-extractable polyphenols, proanthocyanidins with flavanones and ellagitannins are important substances that have anti-inflammatory and antioxidant effects, anticarcinogenic properties and the ability to eliminate free radicals [63]. 

The DPPH analysis measured how many milligrams of sample were needed to reduce the concentration of the DPPH by 50%, so the lower the value, the higher the antioxidant capacity. The extraction with ethanol was more effective, and DPPH values were similar to those reported for CS extracts obtained by treating the coffee silverskin with subcritical water [64]. The CS antioxidant capacity identified by FRAP presented better results for the methanol–acetone solvent combination: approximately three times the value reported in the literature [65] for ethanol extraction. The fact that the most efficient solvent varied between FRAP and DPPH indicates that CS contains antioxidant compounds with different mechanisms of action, suggesting that ethanolic extracts are probably richer in compounds that act by radical quenching. 

##### Cake Characterization 

The addition of CS increased the total dietary fiber content of the cakes (Table 4). There was a significant difference between the samples with and without CS with respect to insoluble dietary fiber content, so that the greater the amount of CS added, the greater the insoluble dietary fiber content was. For soluble fibers, there was no statistical difference between the samples. According to the Brazilian Supplementary Nutrition Information Regulation [58], F1 is considered to be a fiber source because it contains more than 3 g of fiber per 100 g of prepared product, and F3 is classified as high in dietary fiber, containing more than 6 g of fiber per 100 g of prepared product.

Extractable and non-extractable phenolics and total antioxidant capacity (DPPH and FRAP) are displayed in Table 5. The addition of CS increased the extractable and non-extractable phenolics) content of cakes in comparison to those of the cake that did not contain CS. The antioxidant capacity of the cakes, based on either DPPH or FRAP, increased with the addition of CS, without significant differences with respect to the amount of CS added. Other studies have also observed increased antioxidant activity and TEPs when adding CS to other food products, including breads, cakes, yogurt, candies and cookies [44,45,46,47]. It is noteworthy to point out that the DPPH results indicated that the addition of CS nearly doubled the antioxidant activity of the cakes. These results agree with a previous study that employed CS as a fat replacer in cake formulations [50]. However, a direct comparison is not possible because of the differences in terms of cake ingredients and proportions).

Although acrylamide levels were not evaluated in this study, they could be viewed as a concern given the thermal treatment of both CS (roasting) and the prepared cakes (baking). Acrylamide levels have been reported to range between 71 and 81 in cakes [66], and from 490 to 720 μg kg^−1^ in coffee silverskin and its aqueous extract [60]. Benchmark levels established by the European Commission vary significantly depending on the food product, being as low as 50 μg kg^−1^ for wheat bread, 350 μg kg^−1^ for biscuits and as high as 850 μg kg^−1^ for instant coffee [67]. Considering that the maximum amount of CS added to the chocolate cake in this study was below 5% of the cake’s total weight and the fact that cakes are not included among the regulated baking products, it seems that acrylamide levels are low. Nonetheless, one should keep in mind that variations in processing time and temperature have a significant effect on the concentrations of acrylamide compounds [66], and this should be taken into account in future studies.

Caffein levels were not evaluated, because based on data from the literature, we can estimate that the maximum caffein amount in the cakes would be lower than 0.05 mg/100 g, given the usual caffein levels reported for CS [40,41]. Nonetheless, if caffein is of concern in terms of dietary restrictions, water-soluble content could be extracted from CS prior to its use in cake formulations, using the cascade procedure developed by Chemat and collaborators [68]. In this case, the cake fiber content would still be high, but the cake’s antioxidant potential could be diminished because some phenolics would also be extracted. An alternative would be the selective removal of caffein from the water fraction and partial substitution of the milk with antioxidant-enriched water in the cake formulation. For instance, caffein could be removed by increasing the pH and adding dichloromethane. The polyphenols are anionic and not soluble in dichloromethane, so they would remain soluble in water. The water fraction could then be used to add polyphenols to the cake.

##### Sensory Analysis 

Consumer acceptance data for color, aroma, taste, texture and overall impression are shown in Table 6. Considering the color and aroma, there was no significant difference between the samples with and without CS (*p* > 0.05), corresponding to “like moderately” and “like very much” for both attributes. For the samples with addition of CS, color was one of the best accepted attributes, described as attractive, strong and bright.

The acceptability of the attributes using hedonic scale may result in averages that do not differ significantly from each other. Considering this possibility, the internal preference maps consider the individual variability from each consumer, providing small weights for small variances, complementing acceptance test results [69].

**Figure 3:**
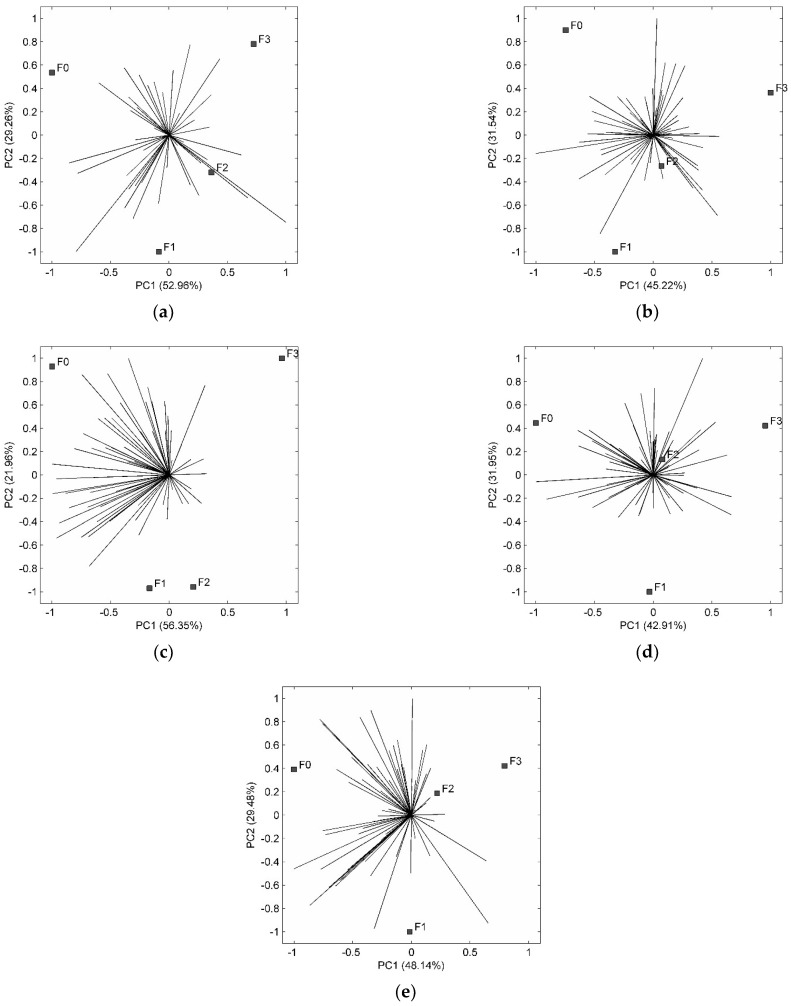
Internal preference mapping for color (**a**), aroma (**b**), taste (**c**), texture (**d**) and overall impression (**e**). PC1: Principal component 1; PC2: Principal component 2.

The internal preference maps for color and aroma are shown in Figure 3a,b, respectively. The two main components explain 82.22% of the variation between the samples for the color and 76.76% for the aroma. Those maps confirm the results of the acceptance test: that the color and aroma of the four samples were equally preferred. Regarding taste (Figure 3c), the cake without CS was the most accepted, with an average of 7.91, corresponding to the hedonic terms “like moderately” and “like very much”. The cakes composed of 2.6% and 3.6% of CS did not differ from each other (*p* > 0.05), and the averages were 6.57 and 6.51, respectively, between the hedonic terms “like slightly” and “like moderately”. In the preference map for taste, the main components explain 78.31% of the variation and the vectors are directed for the cake without CS, which confirms that this cake was the tasters’ favorite. The texture (Figure 3d) differed (*p* < 0.05) only between the cake without CS and the cake containing 2.6% CS, with the first being more accepted. The main components account for 74.86% of the variation in responses, and indicate that the tasters preferred the texture of the cake without CS and with 2.6% and 3.6% of CS (Figure 3c). Although the tasters noted the fibrousness of the cake, they did not consider it to be a disadvantage.

For the overall impression attribute (Figure 3e), all formulations were accepted by the tasters. The hedonic terms for the cakes with CS were “like slightly” and “like moderately”. Through its main components, the preference map (Figure 3e) explains 77.62% of the variation between samples. Considering the samples with CS, the cakes with 2.6% and 3.6% were more preferred.

Our work has demonstrated the wide spectrum of options for sustainable upcycling of coffee silverskin that have been proposed in recent years. However, in the majority of works published so far in the scientific literature, the proposals for upcycling silverskin are focused solely on technical aspects related to processes for transforming such waste into useful products that can be applied in a diverse range of sectors (e.g., food, fuel, cosmetic and pharmaceutical industries). The issue of the economic viability of such processes has not been duly tackled. Most of the silverskin generated in coffee roasters is currently being sent to either composting facilities or landfills, with the latter destination being more common for roasters located within or nearby large urban centers. These methods of disposal incur significant costs for the roaster companies. A significant portion of roasters are micro-branding companies with a relatively small scale of production, in which the volumes of generated silverskin are not large enough to justify their separate collection and transport for low-value use as composting material. Hence, to reduce costs, the implementation of proposals for upcycling unprocessed silverskin should consider the possible uses of such products in commercial establishments near to the roasting company, such as local bakeries. A recent study [70] has demonstrated that valorization of silverskin as a functional ingredient in bakery products would allow coffee roasters to halve their silverskin disposal costs compared to the alternative of sending it to composting facilities. Also, it was concluded from the life cycle analysis that replacing flour with silverskin in bakery products reduces CS’s environmental impact by 96% more than using CS to replace synthetic fertilizers in compost. Therefore, our study on using coffee silverskin as a functional ingredient in cake formulations perfectly fits into this economically viable scenario.

## 4. Conclusions

Coffee silverskin is a by-product generated during coffee roasting. The nutritional profile of CS, especially its abundance in dietary fiber, proteins, and antioxidants, has prompted some studies on its use as an ingredient in food formulations. In this study, it was observed that the CS antioxidant activity and phenolic content (both extractable and non-extractable) presented similar values to fruit peels, which are recognized as sources of antioxidants. The inclusion of CS increased the total dietary fiber content and the antioxidant capacity of chocolate cakes, regardless of the high temperatures achieved during baking. Sensory analysis showed that the cake formulations did not differ in color and aroma, even after the addition of CS. The taste, texture and overall impressions were evaluated; the cake which did not contain CS was preferred by the tasters, with the cake containing 4.6% of CS being the least accepted. Three-way internal preference maps showed that cakes containing 2.6% and 3.6 of CS were equally preferred, and these results indicated that adding up to 3.6% of CS to chocolate cakes can provide a significant improvement in terms of functional potential within acceptable sensory parameters, producing cakes that can be considered as fiber sources. An evaluation of recent studies confirms the potential of CS as a food ingredient for a wide variety of products, but some formulations need to be properly adapted to maintain desirable sensory characteristics.

## Figures and Tables

**Table 1:** Recent developments on potential applications of coffee silverskin.

Area	CS Treatment	Application	Main Results	Ref.
Polymers	Alkaline treatment followed by steam explosion	Reinforcement fibers in geopolymer composites	Enhancement of mechanical and thermal properties of geopolymer composites.	[20]
	Milling and drying followed by dispersion in warm aqueous solution	Additive in wheat flour/glucose-based polymeric films	Increase in surface hydrophobicity, improvement in antioxidant capacity, enhancement of mechanical resistance but decrease in elongation at break.	[21]
	Milling and drying followed by alkali treatment and bleaching	Biocomposite films based on polylactic acid (PLA)	Improvement in mechanical properties and antioxidant capacity	[22]
	Alkaline treatment followed by bleaching	Gel polymer electrolytes for rechargeable zinc-ion batteries	Improvement in mechanical properties of the polymer electrolytes without affecting electrochemical stability.	[23]
Cosmetics	Supercritical CO_2_ extraction	Active ingredient in skin cream	Upregulation of genes involved in oxidative stress responses and skin-barrier functionality; protection of the skin and against Sodium Lauryl Sulfate-induced irritation; improvement in perceived skin hydration.	[24]
Energy	Slow pyrolysis	Biochar	Increased calorific value in comparison to the raw material. Pyrolysis liquid contains caffeine, acetic acid, levulinic acid and formic acid, and thus could serve as a platform for the synthesis of chemicals	[25]
Animal Feed	Grinding followed by mixture with other feed ingredients	Insect larvae feed	CS-based feed enriched with microalgae provided increases in protein and lipid contents of insect (*Hermetia illucens*) larvae	[26]
Soil amendment	Moistening and pressing before storage in sealed plastic bags	Co-composting with gardening prune and biochar	Employment of a 1:1 ratio of CS–co-substrate with extra CS doses added during composting provided the best results; addition of biochar provided adequate porosity conditions for aerobic digestion.	[27]

**Table 2:** Cake formulations.

Ingredient	F0 (Control)	F1 (2.6% CS)	F2 (3.6% CS)	F3 (4.6% CS)
Chocolate cake mixture (g)	400	380	372.5	365
Whole-milk (g)	220	220	220	220
Eggs (g)	145	145	145	145
Coffee silverskin (g)	0	20	27.5	35

**Table 3:** Total extractable phenolics, proanthocyanidins, and antioxidant capacity of coffee silverskin (solvents are indicated in parentheses).

TEP (mg GAE/100 g)	NEPA (mg/100 g)	DPPH IC_50_ (g/g DPPH)	FRAP (µmol Fe_2_SO_4_/g)
783 ± 84 ^a^ (MeAc) 469 ± 17 ^b^ (Eth 60%)	236 ± 4 ^b^ (MeAc) 309 ± 0 ^a^ (Eth)	1727.59 ± 112.06 ^a^ (MeAc) 251.15 ± 13.24 ^b^ (Eth)	170.64 ± 2.98 ^a^ (MeAc) 98.37 ± 1.41 ^b^ (Eth)

Mean ± standard deviation (n = 3). Different letters in the same column indicate that values are significantly different (*p* < 0.05). TEPs = total extractable phenolics; NEPAs = non-extractable phenolics (proanthocyanidins); MeAc (50% methanol; 70% acetone); Eth (ethanol).

**Table 4:** Dietary fiber content of chocolate cakes.

Cake Formulation	Dietary Fiber (Dry Matter Basis) (g/100 g)		
	IDF	SDF	TDF
F0	1.91 ± 0.18 ^c^	0.83 ± 0.14 ^a^	2.74
F1	4.30 ± 0.13 ^b^	1.13 ± 0.16 ^a^	5.43
F3	5.37 ± 0.02 ^a^	0.92 ± 0.34 ^a^	6.29

F0 = cake without CS; F1 = cake with 2.6% CS; F3 = cake with 4.6% CS. Mean ± standard deviation (n = 3). Different letters in the same column indicate that values are significantly different (*p* < 0.05). IDF = insoluble dietary fiber; SDF = soluble dietary fiber; TDF = total dietary fiber.

**Table 5:** Total extractable phenolics, proanthocyanidins (NEPAs) and antioxidant capacity of chocolate cakes.

Cake Formulation	TEPs (mg GAE/100 g) (MeAc)	NEPA * (mg/100 g) (MeAc)	DPPH IC_50_ (g/g DPPH) (MeAc)	FRAP (µmol Fe_2_SO_4_/g) (Eth)
F0	87 ± 2 ^c^	8.89 ± 0.12 ^b^	50,384.9 ± 9302.4 ^b^	2.84 ± 0.19 ^b^
F1	119 ± 3 ^a^	16.96 ± 0.12 ^a^	21,649.8 ± 1892.0 ^a^	6.12 ± 0.56 ^a^
F3	100 ± 2 ^b^	16.11 ± 0.12 ^a^	27,501.2 ± 3935.1 ^a^	6.49 ± 0.11 ^a^

F0 = cake without CS; F1 = cake with 2.6% CS; F3 = cake with 4.6% CS. Mean ± standard deviation n = 5 for TEPs; n = 3 for the others). Different letters in the same column indicate that values are significantly different (*p* < 0.05). TEPs = total extractable phenolics; NEPAs = non-extractable phenolics (proanthocyanidins). Solvents indicated in parentheses: MeAc (50% methanol; 70% acetone); Eth (60% ethanol). * Statistical analysis was conducted via a Kruskal–Wallis non-parametric test.

**Table 6:** Mean scores of cake acceptance for color, smell, taste, texture and overall impression on a 9-point hedonic scale.

Cake Formulation	Color	Smell	Taste	Texture	Overall Impression
F0	7.83 ± 1.10 ^a^	7.4 ± 1.40 ^a^	7.91 ± 0.99 ^a^	7.24 ± 1.55 ^a^	7.61 ± 1.22 ^a^
F1	7.74 ± 1.10 ^a^	7.27 ± 1.36 ^a^	6.58 ± 1.75 ^b^	6.68 ± 1.88 ^b^	6.75 ± 1.56 ^b^
F2	7.71 ± 1.47 ^a^	7.45 ± 1.37 ^a^	6.51 ± 1.91 ^b^	7.14 ± 1.69 ^ab^	6.74 ± 1.72 ^b^
F3	7.61 ± 1.25 ^a^	7.02 ± 1.55 ^a^	5.81 ± 2.21 ^c^	6.89 ± 1.92 ^ab^	6.35 ± 1.84 ^b^

F0 = cake without CS; F1 = cake with 2.6% CS; F2 = cake with 3.6% CS; F3 = cake with 4.6% CS. Mean value ± standard deviation. Mean values were based on the following scores on the hedonic scale: 1: disliked extremely, 2: disliked very much, 3: disliked moderately, 4: disliked slightly, 5: neither liked nor disliked, 6: liked slightly, 7: liked moderately, 8: liked very much, 9: liked extremely. Same letters in the same column indicate that values are not significantly different (*p* > 0.05).

## Data Availability

The original contributions presented in the study are included in the article, further inquiries can be directed to the corresponding author.
